# Immunotherapy using slow-cycling tumor cells prolonged overall survival of tumor-bearing mice

**DOI:** 10.1186/1741-7015-10-172

**Published:** 2012-12-27

**Authors:** Qing Sun, Yong Zhong, Fan Wu, Chunxia Zhou, Dongmei Wang, Wenbo Ma, Youhui Zhang, Shuren Zhang

**Affiliations:** 1 Department of Immunology, Cancer Hospital & Institute, Peking Union Medical College and Chinese Academy of Medical Sciences, Beijing 100021, China; 2 Department of Abdominal Surgery, Cancer Hospital & Institute, Peking Union Medical College and Chinese Academy of Medical Sciences, Beijing 100021, China

**Keywords:** cancer relapse, drug resistance, slow-cycling tumor cells, tumor vaccine

## Abstract

**Background:**

Despite considerable progress in the development of anticancer therapies, there is still a high mortality rate caused by cancer relapse and metastasis. Dormant or slow-cycling residual tumor cells are thought to be a source of tumor relapse and metastasis, and are therefore an obstacle to therapy. In this study, we assessed the drug resistance of tumor cells in mice, and investigated whether vaccination could promote survival.

**Methods:**

The mouse colon carcinoma cell line CT-26 was treated with 5-fluorouracil to assess its sensitivity to drug treatment. Mice with colon tumors were immunized with inactivated slow-cycling CT-26 cells to estimate the efficacy of this vaccine.

**Results:**

We identified a small population of slow-cycling tumor cells in the mouse colon carcinoma CT-26 cell line, which was resistant to conventional chemotherapy. To inhibit tumor recurrence and metastasis more effectively, treatments that selectively target the slow-cycling tumor cells should be developed to complement conventional therapies. We found that drug-treated, slow-cycling tumor cells induced a more intense immune response *in vitro*. Moreover, vaccination with inactivated slow-cycling tumor cells caused a reduction in tumor volume and prolonged the overall survival of tumor-bearing mice.

**Conclusions:**

These findings suggest that targeting of slow-cycling tumor cells application using immunotherapy is a possible treatment to complement traditional antitumor therapy.

## Background

In the majority of cancer cases, mortality is caused by metastases, with only 10% being caused by the primary tumor [1]. In many cancers, metastases and relapses may occur several years or decades after disease remission. Disseminated tumor cells or residual treatment-resistant tumor cells may persist in a so-called dormant state until they are stimulated into an active cell-cycle and initiate tumor recurrence [2]. Thus, these dormant or 'slow-cycling' residual tumor cells are thought to be a source of tumor relapse and metastasis, and are therefore an obstacle to therapy. However, the identification and functional characterization of slow-cycling tumor cells are still poorly understood.

It is accepted that slow-cycling tumor cells are more drug-resistant than normal tumor cells, although direct proof of this is lacking. The suggested mechanism of the drug resistance of slow-cycling tumor cells is that their minimal activity silences a vast spectrum of metabolic loops targeted by anticancer drugs [3]. However, this theory is still controversial, and more research is needed.

Clinical studies have recently shown that adding immunotherapy to chemotherapy has survival benefits compared with chemotherapy alone, and can sensitize tumors to immune-cell-mediated killing [4]. Cancer vaccination with inactivated tumor cells is one form of immunotherapy that is in common use. Studies that have identified slow-cycling tumor cells as the source of tumor relapse and metastasis have also indicated their possible use in cancer vaccination. It is likely that some proteins with distinct immunogenicity are specifically expressed on the surface of slow-cycling tumor cells, which therefore provides opportunities for enhanced immunotherapy.

In the present study, we investigated the tumorigenicity and drug-resistant potential of slow-cycling tumor cells compared with normal tumor cells, and found evidence supporting the hypothesis that slow-cycling, drug-resistant tumor cells are the source of tumor relapse and metastasis, and are thus an obstacle to therapy. We found that, compared with normal tumor cells, the inactivated slow-cycling, drug-resistant cells induced greater proliferation of spleen cells and higher production of interferon (IFN)-γ by these spleen cells *in vitro*. We also investigated the use of such tumor cells in cancer vaccination. We found that vaccination using the slow-cycling, drug-resistant tumor cells induced a conspicuous immune response in mice with colon carcinoma and remarkably prolonged the overall survival of the animals.

## Methods

### Ethics

Experimental research that is reported in the manuscript have been performed with the approval of the Animal Care and Welfare Committee of CIH-CAMS-PUMC (approval date: 20 June 2009; approval number: 20120002). All the experimental research on animals followed the National Institutes of Health *Guide for the Care and Use of Laboratory Animals *(publication no. 85-23, revised 1985).

### Mice

Female 6-week-old Balb/C mice (Animal Center of the Chinese Academy of Medical Sciences, Beijing, China) were kept under specific pathogen-free conditions.

### Cell line and cell culture

All mouse tumor cell lines were cultured in RPMI 1640 medium (Gibco-BRL, Gaithersburg, MD, USA) supplemented with 10% FBS, at 37°C in a humidified atmosphere containing 5% CO_2_. YAC-1: a mouse lymphoma cell line which is a specific target for NK cells. We used mouse TC-1 tumor cells derived from primary epithelial cells of C57BL/6 mice co-transformed with HPV-16 E6, E7 and c-Ha-ras oncogene (kind gift of Dr TC Wu, Johns Hopkins Medical Institutions, Baltimore, MD, USA); 4T1 (a mammary gland tumor cell line from Balb/C mice with high metastatic potency); and CT-26 (a colon tumor cell line from Balb/C mice) (both American Type Culture Collection (ATCC), Manassas, VA, USA).

### DiI staining and cell sorting

Tumor cells were stained with DiI (Dil (1,1'-dioctadecyl 3,3,3',3'-tetramethyl-indocarbocyanine perchlorate); Invitrogen Corp., Carlsbad, CA, USA) in accordance with the protocol for attached cells [5]. Cells were suspended at a density of 1 × 10^6^/ml in 1640 culture medium, DiI solution was added at a concentration of 5 μl/ml and the cell suspension was incubated at 37°C for 20 minutes. After washing with phosphate-buffered solution (PBS) with 2% FBS, the cells were analyzed and sorted using a fluorescence-activated cell sorting (FACS) system (Vantage SE; Becton Dickinson, Franklin Lakes, NJ, USA).

To determine the heterogeneity of tumor cells with respect to cell-cycle length *in vitro*, DiI-labeled cells were allowed to grow for 8 days in complete RPMI 1640 culture medium under normal conditions, and were analyzed by flow cytometry on days 1, 3, 5, and 8. To determine the heterogeneity of tumor cells with respect to cell-cycle length *in vivo*, DiI-labeled cells were injected subcutaneously into the left groin of Balb/C mice (2 × 10^6 ^cells per mouse; four mice in total). Tumors were digested in complete RPMI medium containing 1 mg/ml type IV collagenase and 300 U/ml DNase I (Sigma AB, Göteborg. Sweden) incubated for 30 min at 37°C, and analyzed by flow cytometry on days 10, 15, and 25. When sorting, the slow-cycling cells were identified as a bright positive population.

### Hoechst-Pyronin Y staining and cell sorting

Cells were detached from the cell-culture flask with 0.1% trypsin, and Trypan blue-nonstaining viable cells were counted and suspended at a density of 1 × 10^6^/ml in DMEM culture medium. Then they were stained with the fluorescent dye Hoechst 33342 (Sigma AB) at a concentration of 5 μg/ml at 37°C for 45 minutes. At the end of this time, 1 μg/ml of Pyronin Y (PY) was added, and cells were incubated at 37°C for an additional 45 minutes as described previously [6]. After washing with PBS plus 2% FBS, the cells were analyzed and sorted (FACS Vantage SE; Becton Dickinson). When sorting, cells residing in the G0/G1 peak that simultaneously stained weakly with PY were regarded as cells in G0 phase, and these were sorted and used for further studies [7].

### Side-population analysis

Cells were detached from the cell-culture flask with 0.1% trypsin, and Trypan blue-nonstaining viable cells were counted, and suspended at a density of 1 × 10^6^/ml in DMEM culture medium. Then they were stained with the fluorescent dye Hoechst 33342 (Sigma AB) at a concentration of 5 μg/ml (37°C for 90 min) as described previously [8]. After washing with PBS plus 2% FBS, the cells were incubated with 2 μg/ml propidium iodide (PI) to exclude dead cells, then cell analysis was performed (FACS Vantage SE; Becton Dickinson).

### Tumor generation

Viable fast-cycling and slow-cycling tumor cells obtained using the DiI-based FACS, and viable G0 and non-G0 cells obtained by Hoechst-PY-based FACS, were stained with Trypan blue and counted. Then cells of every population were injected subcutaneously into the left groin of Balb/C mice at a gradient dose of 5000, 1000, or 500 cells. The mice were examined visually every day.

### Chemotherapy resistant assay

To investigate the chemotherapy resistance of slow-cycling cells *in vivo*, DiI-labeled cells (1 × 10^6 ^per mice) were injected subcutaneously into Balb/C mice. When the tumors had grown to 10 × 10 mm in size, 5-fluorouracil (5-FU) 40 mg/kg was injected intraperitoneally every 3 days for a total of four injections. The vehicle control mice were injected with PBS, using the same regimen. After the final treatment, tumors were digested into a single-cell suspension as described above, and analyzed by flow cytometry the next day.

To determine the chemotherapy resistance of slow-cycling cells *in vitro*, the same numbers of DiI-labeled cells were seeded into a cell-culture flask (day 1), and grown for 24 hours, then treated with 5-FU (day 2) at a concentration of 1.5 μg/ml. On day 3, the medium was replaced with fresh medium without 5-FU, and the cells were grown under normal conditions for 24 hours. On day 4, 5-FU 1.5 μg/ml was added into the medium again, and cells were grown for a further 24 hours, then on day 5, the medium was again replaced with fresh medium without 5-FU, and cells were grown for another 24 hours. Finally, on day 6, cells were treated with trypsin and analyzed by flow cytometry. The control cells were treated in the same way but were never exposed to 5-FU.

To detect the inhibition of cell proliferation by 5-FU *in vitro*, DiI-labeled cells of test group and control group were seeded in triplicate into 96-well culture plate at 3,000 cells/well, then challenged with 5-FU 24 hours later in the same manner above. On day 6, 3-(4,5-dimethylthiazol-2-yl)- 2,5- diphenyltetrazolium bromide (MTT) method was performed as described previously [9].

### *In vitro *lymphocyte proliferation assay

Mixed lymphocyte tumor cell culture (MLTC) was used to investigate the proliferation of spleen cells. Tumor-bearing mice were killed by broken neck and spleens were harvested. The spleen tissue was ground and suspended in PBS, then spleen cells were isolated using density gradient centrifugation (Ficoll-Hypaque, Haoyang Biological Manufacture, Tianjin, China) and stored as a single-cell suspension. CT-26 cells treated with 5-FU (FU-CT-26) or not (non-FU-CT-26) were exposed to mitomycin C (MMC) for 1.5 hours, then these cells were seeded in triplicate at a density of 1 × 10^4 ^cells per well in 96-well culture plates, along with spleen cells (1 × 10^5 ^cells per well), and incubated with interleukin (IL)-2 (100 U/ml) for 4 days at 37°C in a humidified 5% CO_2 _atmosphere. The MTT (3-(4,5-dimethylthiazol-2-yl)-2,5-diphenyltetrazolium bromide) assay was used to test the lymphocyte proliferation [9], and the results were expressed as:

$$\text{proliferation}\;\text{index}=\left[\left(\text{A}-\text{B}\right)/\text{C}\right]\times 100\%,$$

where A is the experimental absorbance from the spleen plus tumor cell co-cultures, B is the absorbance from the tumor cells alone, and C is the absorbance from the spleen cells alone.

### ELISA for the production of interferon-gamma (IFN-γ)

CT-26 cells treated with 5-FU or not were exposed to MMC for 1.5 hours, then these cells (2 × 10^5 ^cells per well) were co-cultured separately with spleen cells (2 × 10^6 ^cells per well) from tumor-bearing mice at ratio of 1:10 in 400 μl complete RPMI 1640 containing IL-2 (100 U/ml) for 3 days. The supernatant was collected on day 4, and the concentration of IFN-γ was analyzed using a mouse IFN-γ ELISA kit (eBioscience Inc., San Diego, CA, USA) as described previously [10].

### Flow cytometry and antibodies

The following anti-mouse monoclonal antibodies (mAbs) were used for flow cytometry: anti-H2-Kd-PE (phycoerythrin conjugated); anti-major histocompatibility complex (MHC) I-PE-Cy5; anti-CD80-FITC (fluorescein isothiocyanate) and anti-CD86-FITC (BioLegend, San Diego, CA, USA). Flow cytometry was performed using a flow cytometer (Epics XL; Beckman Coulter Inc., Brea, CA, USA) equipped with Expo32 software (Beckman Coulter).

### *In vitro *cytotoxic assay

Mice (three mice per group, and three groups in total) were challenged with 3 × 10^5 ^CT-26 cells injected subcutaneously into the left groin (day 0), then separately immunized with subcutaneous injection of FU or non-FU-CT-26 cells (1 × 10^6^) that had also been pretreated with MMC on days 3, 6, 9, 13, 18, and 25. 7 days after the final booster. Spleen cells from the immunized mice (FU or non-FU-CT-26 groups) were prepared as effector cells. Mice in the control group were treated in the same way, but using PBS for injection.

4T-1, YAC-1, and FU or non-FU CT-26 cells were used as target cells. As described previously [11], target cells were labeled with 5- (and 6-) carboxyfluorescein diacetate succinimidyl ester (CFSE; Sigma AB) for 10 minutes at 37°C using a final concentration of 2 μmol/L. After labeling, the cells were washed once and re-suspended in complete RPMI 1640. Effector and target cells were mixed to a final volume of 200 μl in complete RPMI 1640, with the ratio of effector:target being 50:1. The tubes were mixed and spun down at 120 × g for 2 minutes, then the samples were incubated at 37°C for 4 hours. At the end of incubation time, 2.5 μg PI (Sigma AB) was added for DNA labeling of dead cells. The samples were then incubated for 5 minutes and analyzed by flow cytometry within 60 minutes.

### Vaccination treatment in a murine colon cancer model

To establish a colon cancer model, 3 × 10^5 ^CT-26 cells were subcutaneously inoculated at the left groin of Balb/C mice on day 0 (five mice per group, and five groups in total). Then tumor cells were injected combined with or not with granulocyte-macrophage colony-stimulating factor (GM-CSF, 1 ng per mouse) on days 3, 6, 9, 13, 18, 25. The FU and non-FU CT-26 cells used in the earlier vaccination were pretreated with MMC and injected subcutaneously at 1 × 10^6 ^per mouse. Control mice were treated with PBS. Tumor growth was monitored every 2-3 days by palpation, and tumor size was measured in two perpendicular tumor diameters, as described previously [12].

### Statistical analysis

Statistical significance of difference between the two groups was determined by the Student paired t-test. The Kaplan-Meier plot for survival was assessed for significance using the log-rank test (SPSS software; version 12.0; SPSS Inc., Chicago, IL, USA). *P *< 0.05 was considered significant.

## Results

### Tumor cells exhibit clear heterogeneity in to cell-cycle length both *in vivo *and *in vitro*

**Figure 1:**
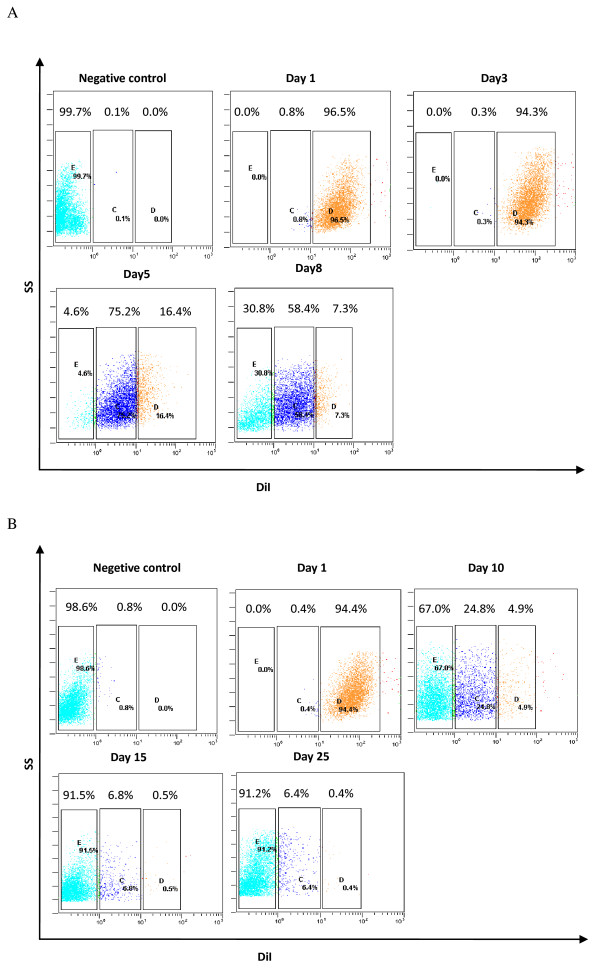
**The DiI-tracing assay showed that tumor cells exhibited clear heterogeneity with respect to cell-cycle length**. **(A) **DiI-labeled CT-26 cells were cultured in complete RPMI 1640 medium on day 1, and the percentage of DiI-retaining cells was analyzed on days 1, 3, 5, and 8 by flow cytometry. **(B) **DiI-labeled CT-26 cells were injected subcutaneously into mice on day 1. On days 10, 15 and 25, tumors were excised and digested, and the percentage of DiI-retaining cells was analyzed by flow cytometry. Experiments were repeated twice with similar results.

DiI is a long-term lipophilic tracer dye that can be used to trace cell division and identify slow-cycling cells. It has several advantages compared with PKH26, such as a simpler protocol for cell labeling, lower cytotoxicity, and higher resistance to intercellular transfer [13]. This dye normally disappears at cell division; however, slow-cycling cells may retain it for a long time, which allows them to be identified by flow cytometry. We found that DiI-retaining cells disappeared when they were allowed to grow *in vitro *or *in vivo *(Figure 1A, B). A bright DiI-positive population of cells (DiI^high^) was visible after 8 days in culture (7.3% of cells; Figure 1A) or 25 days growing *in vivo *(0.5% of cells; Figure 1B). Tumor cells exhibited clear heterogeneity with respect to cell-cycle length both *in vivo *and *in vitro*, with slow-cycling tumor cells (DiI^high^), cycling tumor (DiI^low^) cells, and fast-cycling tumor cells (DiI-negative cells) all being present. Moreover, slow-cycling tumor cells only comprised a small proportion of the tumor mass.

### Slow-cycling tumor cells have the character of stem cells

Slow-cycling tumor cells are thought to be drug-resistant and the source of relapse and metastasis. To investigate the tumorigenic potential of this cell population, freshly sorted slow-cycling and fast-cycling tumor cells were injected subcutaneously into Balb/C mice, and their tumor-formation ability was assessed.

A bright DiI-retaining population of cells was selected as slow-cycling cells, and a population of DiI-negative cells was selected as fast-cycling cells. After injection of 5,000 DiI^high ^or DiI-negative cells, all five mice that received the DiI^high ^cells developed tumors, whereas only three of five mice that received DiI-negative cells developed tumors. Similarly, when the number of cells injected decreased to 1000, tumors formed in three of five mice that received DiI^high ^cells, whereas tumors were only seen in one of five mice injected with the DiI-negative cells. After injection of 500 DiI^high ^cells, only one of five mice had established tumors, whereas no tumors were established from DiI-negative cells (Table 1).

**Table 1:** Tumor-generation assay after using injection of 500, 1,000 or 5,000 DiI-positive cells^a, b^.

	Tumor generation, n/total n			Mean time to tumor generation, days		
cell injection	5,000^c^	1,000^d^	5,00^d^	5000	1000	500

DiI^high ^cells	5/5	3/5	1/5	28	29	37
DiI^- ^cells	3/5	1/5	0/5	21	24	--

^a^Experiments were repeated three times with similar results; this table presents the result of one experiment.

^b ^DiI^high ^cells were isolated using DiI-based fluorescence-activated cell sorting.

^c, d^The *t*-test was carried out to determine the difference between the two groups in three experiments: ^c^*P *< 0.01; ^d^*P *< 0.05,).

**Figure 2:**
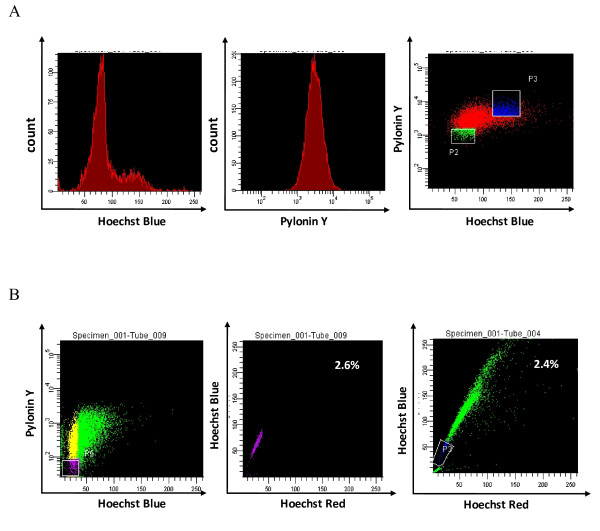
**Most cells in the G0 phase sorted by fluorescence-activated cell sorting (FACS) also resided in the same region as side-population cells**. **(A) **Hoechst-Pyronin Y (PY)-based FACS. The left and middle panels separately represent the Hoechst-staining control group and PY-staining control group. In the Hoechst-PY-staining group (right), the P2 population represents the cells residing in G0 phase, and P3, cells in S phase. **(B) **G0 cells sorted by Hoechst-PY-staining-based FACS (left panel) were measured by side-population analysis, and the result is shown in the middle panel. The right panel represents the classic side-population analysis of CT-26 cells. The middle and right panels show that most of the cells in G0 phase also resided in the region of the side-population cells. Experiments were repeated three times with similar results.

Analogous results were seen in cells sorted by Hoechst-PY stain-based FACS. There are many dyes available for determining G0/G1 versus S and G2/M phases based on the DNA content measured by flow cytometry. However, a DNA dye is not able to distinguish cells residing in G0 or G1 phases. This can be achieved by quantifying RNA content (which increases during G1 and remains high during mitosis) using PY in conjunction with the DNA dye Hoeschst 33342 [14]. Tumor cells residing in the G0/G1 peak and simultaneously weakly stained by PY were sorted as G0 cells (P2; Figure 2A), whereas tumor cells residing in S phase were regarded as non-G0 cells (P3; Figure 2A). The results of the tumor-generation assay were similar to those of the assay mentioned in the previous paragraph. Tumors formed in all five mice injected with 5,000 G0 cells, compared with two of five mice injected with 5,000 non-G0 cells (Table 2); in all five mice injected with 1,000 G0 cells, compared with only one of five mice injected with 1,000 non-G0 cells; and in three of five mice injected with 500 G0 cells, compared with none of the mice injected with non-G0 cells. All these results indicated increased tumorigenicity of slow-cycling tumor cells.

**Table 2:** Tumor-generation assay using injection of 500, 1,000 or 5,000 G0 cells^a, b^.

	Tumor generation, n/total n			Mean time to tumor generation, days		
cell injection	5,000^c^	1,000^c^	500^c^	5,000	1,000	500

G0	5/5	5/5	3/5	25.4	26.6	27.6
Non-G0	2/5	1/5	0/5	22	21	--

^a, b^

^a^Experiments were repeated three times with similar results; this table presents the result of one experiment.

^b^G0 cells were isolated using Hoechst-Pyronin Y-based fluorescence-activated cell sorting.

^c^The *t*-test was carried out to determine the difference between the two groups in three experiments: ^c^*P *< 0.01.

Until recently, side-population analysis has been one of the accepted methods for identifying cancer stem cells (CSCs) [15]. We investigated the percentage of side populations in CT-26 cells (Figure 2B, right). We then analyzed the number of G0 cells sorted by Hoechst-PY staining-based FACS (Figure 2B, left) using side-population analysis (Figure 2B, middle). G0 cells resided in almost the same part of the coordinate axis (Figure 2B, middle) as the side-population cells (Figure 2B, right), and they had a similar proportion of tumor cells (2.6% versus 2.4%; Figure 2B, middle and right). These findings and the increased tumorigenicity of slow-cycling tumor cells indicated that they might have the character of CSCs.

We found that although slow-cycling tumor cells exhibited a higher tumorigenic potential, the average number of days of tumor generation was prolonged compared with that of fast-cycling cells (Table 1, Table 2).

### Slow-cycling tumor cells are more resistant to chemotherapy, both *in vivo *and *in vitro*

Slow-cycling tumor cells have always been predicted to be resistant to traditional chemotherapy. We treated CT-26 cells with 5-FU *in vivo *and *in vitro *to investigate the sensitivity of slow-cycling cells and normal tumor cells.

**Figure 3:**
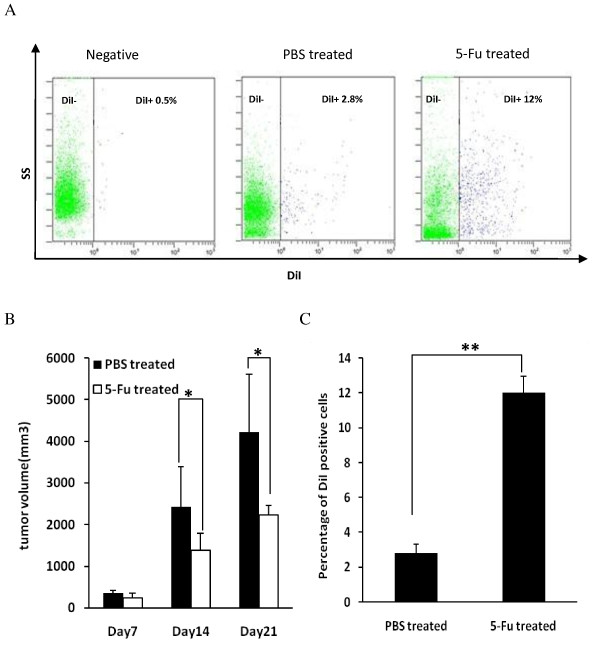
**Chemotherapy-induced enrichment of DiI-retaining cells *in vivo***. **(A) **DiI-retaining tumor cells were enriched *in vivo *after treatment with 5-fluorouracil (FU). Negative: flow cytometry of CT-26 cells that were not stained with DiI: PBS-treated: flow cytometry of cells from tumor treated with phosphate-buffered saline (PBS); 5-FU-treated: flow cytometry of cells from tumor treated with 5-FU. **(B) **Tumor volume in mice after chemotherapy compared with the PBS-treated group (*P < 0.05, *t*-test). **(C) **Percentage of DiI-positive cells in tumors with or without chemotherapy (**P < 0.01, *t*-test). Error bars represent the standard deviation. DiI-labeled CT-26 cells were injected subcutaneously into Balb/C mice on day 1. When tumors grew to 10 × 10 mm, 5-FU 40 mg/kg was injected intraperitoneally every 3 days for a total of four injections. Vehicle-treated control mice were exposed to the same regimen, but injected only with PBS. Tumor volume was measured on days 7, 14, and 21. After the final treatment, tumors were digested and analyzed by flow cytometry on the next day. Experiments were repeated twice with similar results.

Tumor volume in mice that received chemotherapy decreased markedly compared with that in PBS-treated mice (Figure 3B). However, after four treatments with 5-FU, the percentage of DiI-retaining slow-cycling cells increased significantly, being 12% in tumors from mice that received chemotherapy compared with 2.8% in tumors from mice treated with PBS (Figure 3A, C).

**Figure 4:**
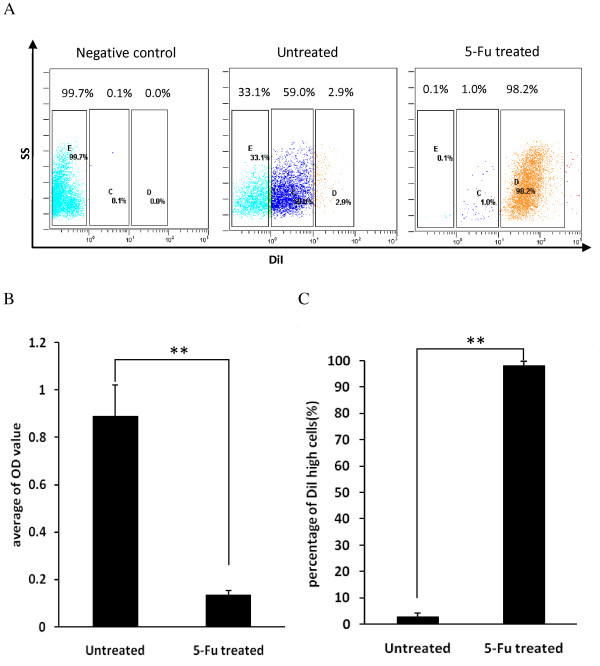
**Chemotherapy-induced enrichment of DiI-retaining cells *in vitro***. **(A) **DiI-retaining tumor cells were enriched *in vitro *after treatment with 5-fluorouracil (FU). DiI-labeled CT-26 cells were cultured in complete RPMI 1640 medium and exposed to 5-FU on day 2. The old medium was changed for fresh medium without 5-FU on day 3, then 5-FU was added again on day 4. Cells were harvested on day 6 and analyzed by flow cytometry. The control group was cultured without 5-FU. **(B) **Persistence of cells after 5-FU treatment. The same numbers of CT-26 cells were seeded into 96-microwell plates. The 5-FU-treated group was treated as described above, and the untreated group was cultured without 5-FU. An MTT assay was performed 5 days later. (**P < 0.01, *t*-test) **(C) **Percentage of DiI-positive cells *in vitro *after 5-FU treatment. (**P < 0.01, *t*-test). Experiments were repeated twice times with similar results. Error bars represent the SD.

Similarly, *in vitro*, 5-FU treatment obviously inhibited the proliferation of tumor cells, as indicated by the absorbance of MTT (Figure 4B). Tumor cells after chemotherapy were analyzed by flow cytometry; most cells were DiI-retaining slow-cycling cells (98.2%), while the percentage of DiL-positive cells in the untreated group was only 2.8% (Figure 4A, C). These findings were consistent with the previous prediction that slow-cycling tumor cells are an obstacle to traditional chemotherapy.

### 5-fluorouracil-treated CT-26 cells induce increased proliferation of and interferon-γ production by spleen cells *in vitro*

**Figure 5:**
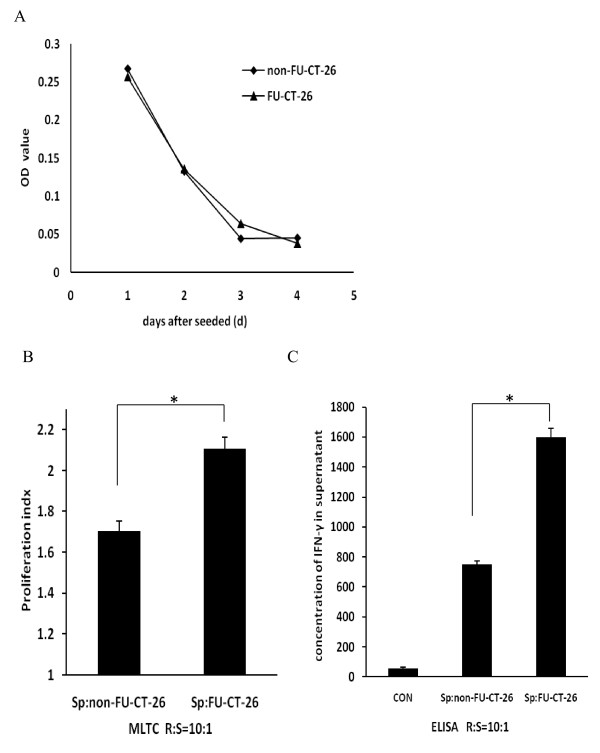
**5-fluorouracil (FU)-treated CT-26 cells induced increased proliferation and interferon (IFN)-γ production by spleen cells**. **(A) **Proliferation curve of FU-treated and non-FU-treated CT-26 cells after treatment with mitomycin C (MMC). No proliferation was seen with either of the tumor cells, which confirmed the efficacy of MMC. **(B) **Spleen cells from tumor-bearing mice had significantly higher proliferation after co-culture with FU-CT-26 cells compared with non-FU-CT-26 cells, at a responder:stimulator (R:S) ratio of 10:1. (*P < 0.05, *t*-test) **(C) **IFN-γ production by spleen cells after co-culture with 5FU-treated CT-26 cells compared with CT-26 cells. (*P < 0.05, *t*-test). Error bars represent the standard deviation. CON: control spleen cells cultured without tumor cells. Mixed lymphocyte tumor cell culture (MLTC) was performed to investigate the proliferation of and IFN-γ production by spleen cells. Spleen cells were harvested from tumor-bearing Balb/C mice. The same numbers of FU-CT-26 and non-FU-CT-26 cells were seeded into 96-microwell plates. Spleen cells were added at an R:S ratio of 10:1 on day 1, and proliferation of spleen cells was analyzed by MTT assay on day 4. As for IFN-γ production, the same numbers of FU-CT-26 and non-FU-CT-26 cells were seeded into 24-well plates. Spleen cells were added at an R:S ratio of 10:1. The supernatant was collected on day 4, and IFN-γ concentration was analyzed by ELISA.

To investigate the potential of inducing proliferation of and IFN-γ production by spleen cells *in vitro*, FU and non-FU CT-26 cells were treated with MMC and then co-cultured with freshly isolated spleen cells in medium containing IL-2. After 3 or 4 days culture, an MTT assay was performed to analyze the proliferation rate. To investigate the efficacy of MMC, the proliferation of MMC-treated FU-CT-26 and non-FU-CT-26 cells was analyzed. No proliferation was seen in either group of tumor cells after treatment with MMC, which indicates that MMC could be used to inactivate both FU and non-FU CT-26 cells (Figure 5B). The FU-CT-26 cells had a higher proliferation index than the non-FU-CT-26 cells (2.11 versus. 1.70; Figure 5B).

Culture supernatant was collected and used for IFN-γ analysis by ELISA. FU-CT-26 cells induced more IFN-γ production in spleen cells compared with CT-26 cells (Figure 5C). The concentrations of IFN-γ in the supernatant of the FU-CT-26 plus spleen cell mixture was 1602 ± 55, that of the non-FU-CT-26 plus spleen cells was 750 ± 24, and that of the spleen cells alone was 54 ± 11 pg/ml, respectively.

In conclusion, compared with non-FU-CT-26 cells, FU-CT-26 cells induced a more intense immune response.

### Immunotherapy with inactivated fluorouracil-treated CT-26 cells plus granulocyte-macrophage colony-stimulating factor produces a therapeutic effect in a CT-26 colon cancer mouse model

**Figure 6:**
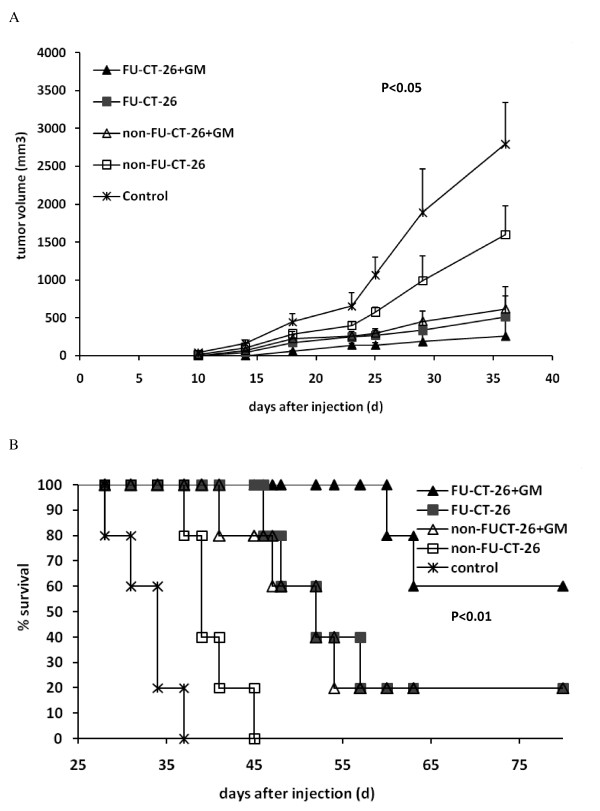
**Therapeutic efficacy of inactivated 5-fluoracil-treated CT-26 cells plus granulocyte-macrophage colony stimulating factor (GM-CSF) against subcutaneous CT-26 tumor in Balb/C mice**. **(A) **Tumor volume of mice in the FU-CT-26 + GM-CSF group decreased significantly compared with other groups. (*P *< 0.05, ANOVA) Error bars represent the standard deviation. **(B) **Kaplan-Meier survival analysis shows that mice treated with inactivated FU-CT-26 cells combined with GM-CSF had longer survival than other groups (P < 0.01). CT-26 cells (10^6) ^were injected subcutaneously into Balb/C mice on day 0. Tumor-bearing mice were vaccinated subcutaneously on days 3, 6, 9, 13, 18, and 25 with different cell vaccines. FU-CT-26+GM: mice immunized with mitomycin C (MMC)-inactivated FU-CT-26 cells (10^6^) plus GM-CSF. FU-CT-26: mice immunized with MMC-inactivated FU-treated CT-26 cells (10^6^). CT-26+GM: mice immunized with MMC-inactivated CT-26 cells (10^6^) plus GM-CSF. CT-26: mice immunized with MMC-inactivated CT-26 cells (10^6^). Control: mice treated with phosphate-buffered saline. Experiments were repeated three times with similar results.

The preceding results provided strong information that slow-cycling tumor cells may be a better immunogen that could induce an intense antitumor response. To investigate whether immunization with inactivated FU-CT-26 cells could obtain a better therapeutic effect *in vivo*, we established a subcutaneous CT-26 mouse model and immunized them separately with inactivated CT-26 and FU-CT-26 combined with GM-CSF. Tumors in mice treated with FU-CT-26 or non-FU-CT-26 cells plus GM-CSF, or non-FU-CT-26 cells alone were all clearly reduced compared with those in mice treated with PBS (control group). Immunization with the FU-CT-26 cells plus GM-CSF produced the best therapeutic effect (Figure 6A). Treatment with FU-CT-26 cells plus GM-CSF prolonged survival of tumor-bearing mice (Figure 6B). Both the mice treated with PBS and the mice treated with the non-FU-CT-26 cells all died, with a median survival of 32.8 and 40.2 days, respectively. The mice immunized with FU-CT-26 and non-FU-CT-26 cells plus GM-CSF had comparable results, with median survival of 50.7 and 48.5 days, respectively. When survival was monitored for up to 80 days after inoculation, one mouse in each group was still alive. However, treatment with FU- CT-26 cells plus GM-CSF exhibited the best outcome, with a median survival of 61.5 days, and a 60% survival rate (three of five mice) when survival was monitored up to 80 days after inoculation.

We rechallenged the mice that survived after immunotherapy (one treated with FU-CT-26 cells alone, one with non-FU-CT-26 cells plus GM-CSF, and three with FU-CT-26 cells plus GM-CSF) with 10^6 ^non-FU-CT-26 cells. None of these five surviving mice had established tumors, which indicated that immunotherapy with inactivated FU-CT-26 and non-FU-CT-26 cells induced a specific memory immune response *in vivo*.

### Immunocytes activated by 5-fluorouracil (FU) CT-26 cells show specific and intense cytotoxicity to FU-CT-26 and non-FU-CT-26 cells

**Figure 7:**
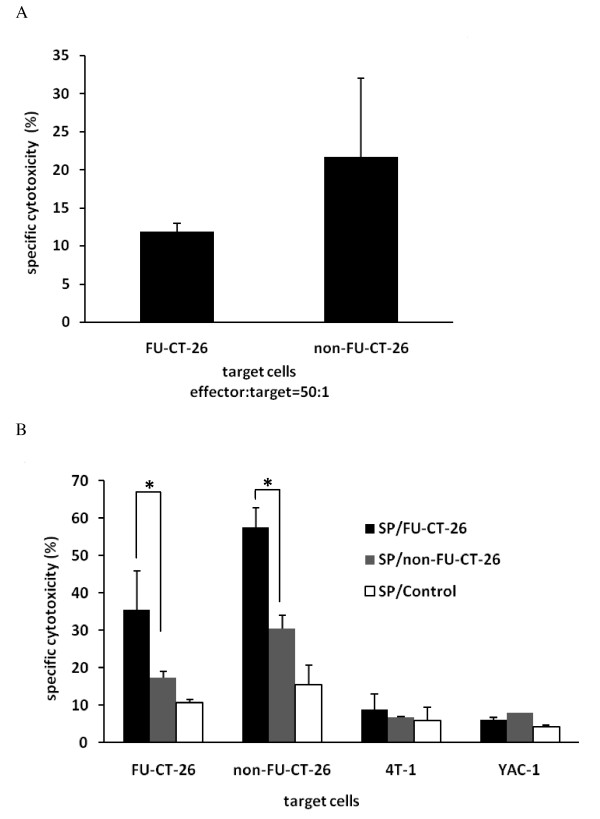
**Cytotoxicity analysis using carboxyfluorescein diacetate succinimidyl estercarboxyfluorescein diacetate succinimidyl ester (CFSE)-propidium iodide (PI) staining-based Flow cytometry**. CFSE- and PI-positive target cells represent cells lysed by effector cells. **(A) **Spleen cells from tumor-bearing mice killed fewer slow-cycling and drug-resistant 5-fluorouracil (FU)-treated CT-26 tumor cells. No significant differences were seen (*t*-test). **(B) **Spleen cells from tumor-bearing mice immunized with inactivated FU-CT-26 or non-FU-CT-26 cells showed tumor-specific cytotoxicity *in vitro*, and spleen cells from mice immunized with inactivated FU-CT-26 cells exhibited higher cytotoxicity compared with the other two groups. (*P < 0.05, **P < 0.01, *t*-test) Error bars represent the standard deviation. From left to right, the target cells respectively were FU-CT-26, non-FU-CT-26, 4T-1, and YAC-1 cells. Effector:target cell ratio was 50:1. SP/FU-CT-26: Spleen cells from tumor-bearing mice immunized with mitomycin C (MMC)-inactivated FU-CT-26 cells. SP/non-FU-CT-26: Spleen cells from tumor-bearing mice immunized with MMC-inactivated CT-26 cells. SP/Control: Spleen cells from tumor-bearing mice treated with PBS. Experiments were repeated three times with similar results.

Immunization with inactivated FU-CT-26 cells showed a clear therapeutic effect *in vivo*, therefore, we investigated the mechanism involved. First, we investigated the sensitivity of slow-cycling tumor cells to killing by cytotoxic T lymphocytes (CTLs). Spleen cells from tumor-bearing mice were isolated, and their cytotoxicity against FU-CT-26 and non-FU-CT-26 cells was analyzed *in vitro*. CTLs showed 11.85% cytotoxicity against FU-CT-26 cells compared with 21.67% cytotoxicity against non-FU-CT-26 cells (Figure 7A). These data indicate that slow-cycling, drug-resistant tumor cells were also resistant to cytotoxic killing, and this coincided with the clinical data.

Second, we investigated whether immunocytes activated by FU-CT-26 cells exhibited specific and obvious cytotoxicity against normal and slow-cycling tumor cells. The cytotoxicity of the induced spleen cells immunized with inactivated non-FU-CT-26 and FU-CT-26 cells was evaluated against a series of cells by flow cytometry. Immunization with FU or non-FU CT-26 cells induced tumor-specific cytotoxicity; the cytotoxicity against 4T-1 and YAC-1 cells was less than 10%, whereas the cytotoxicity against FU and non-FU CT-26 cells was much higher (Figure 7B). Moreover, compared with spleen cells from mice immunized with non-FU-CT-26 cells (SP/non-FU-CT-26), spleen cells from mice immunized with FU-CT-26 cells (SP/FU-CT-26) exhibited higher cytotoxic potential against non-FU-CT-26 cells (SP/FU-CT-26:57 ± 5% versus SP/non-FU-CT-26: 30 ± 3%) and FU-CT-26 cells (SP/FU-CT-26: 35 ± 10% versus SP/non-FU-CT-26:17 ± 1%).

### Upregulation of major histocompatibility complex and co-stimulatory molecules on the surface of fluorouracil-treated CT-26 cells

Using flow cytometry, we analyzed the expression of MHC class I and II molecules and co-stimulatory molecules on the surface of FU and non-FU CT-26 cells. Although the expression of MHC class I molecules was comparable in both FU and non-FU cells (92.7 ± 2.76% versus 99.1 ± 1.27%), the average fluorescence intensity of MHC class I expressed by the FU-CT-26 cells was much lower (13.6 ± 0.21 versus 38.9 ± 2.34). However, compared with the non-FU-CT-26 cells, the expression level and average fluorescence intensity of MHC class II molecules and of the co-stimulatory molecules CD80 and CD86 on the surface of FU-CT-26 cells were all clearly upregulated (Table 3), although the expression level of MHC class II molecules and CD86 was not high.

**Table 3:** Expression levels and fluorescence intensity of MHC and co-stimulatory molecules on the surface of 5-FU-treated cells^a, b^.

Cell type	Percentage of molecule expression, %				Average fluorescence intensity			
	
	MHC I	MHC II	CD80	CD86	MHC I	MHC II	CD80	CD86
CT-26								
Treated	92.7 ± 2.76	**10.0 **± 1.56^c^	92.4 ± 7.85	**9.2 **± 1.84^c^	**13.6 **± 0.21^c^	**12.5 **± 0.42^d^	**4.3 **± 0.14^c^	**3.5 **± 0.49^c^
Untreated	99.1 ± 1.27	1.5 ± 0.14	83.4 ± 6.58	3.1 ± 2.47	38.9 ± 2.34	5.0 ± 0.49	2.7 ± 0.35	1.8 ± 0.64
TC-1								
Treated	92.4 ± 2.69	1.6 ± 0.49	95.9 ± 4.03	**5.3 **± 0.10^c^	**3.0 **± 0.35^c^	**3.6 **± 0.92^c^	**10.7 **± 0.99^c^	2.9 ± 1.06
Untreated	97.5 ± 2.47	0.4 ± 0.07	95.0 ± 1.27	1.6 ± 0.49	8.2 ± 0.78	2.3 ± 0.64	5.6 ± 0.14	1.8 ± 0.71
4T-1								
Treated	69.7 ± 6.65	**10.5 **± 1.13^d^	**11.3 **± 1.27^c^	**7.8 **± 1.27^c^	**2.6 **± 0.49^c^	**5.4 **± 0.49^c^	4.4 ± 0.85	3.5 ± 1.06
Untreated	84.4 ± 0.64	2.4 ± 1.20	2.7 ± 0.71	1.8 ± 0.07	3.8 ± 0.07	3.2 ± 0.64	2.7 ± 1.13	2.4 ± 0.92

Abbreviations: MHC, major histocompatibility complex.

^a^Experiments were repeated twice with similar results.

^b^Data are expressed as mean ± SD of the two independent experiments.

^c, d^The *t*-test was carried out to determine the difference between the two groups in three experiments: ^c^P < 0.05, ^d^P < 0.01, *t*-test).

To confirm this finding, we analyzed the expression of the molecules on the surface of 5-FU-treated 4T-1 and TC-1 or untreated 4T-1 and TC-1 tumor cells by flow cytometry, and a similar tendency was seen (Table 3). These findings indicate that increased expression of MHC class II molecules and the co-stimulatory molecules CD80 and CD86 may be the reason why drug-treated tumor cells can induce a more significant immune response, and lower expression of MHC class I molecules could be one reason for the resistance of slow-cycling tumor cells to cytotoxic killing.

## Discussion

Tumor dormancy has been recognized for many years as a clinical phenomenon in several types of cancer. Clinicians and experimental biologists have used the term 'dormancy' to describe the hypothetical state of cancer cells lying in wait for some time after treatment of the primary tumor, before the tumor's subsequent growth and clinical recurrence [2,16]. Tumors in dormancy are mainly constructed of quiescent or slow-cycling tumor cells. Quiescent tumor cells can be detected in the marrow of many patients in the tumor-remission phase, and these patients often develop tumor relapse or metastasis [17-19]. However, there is insufficient evidence to prove that these cells are the origin of tumor relapse. Thus, more research into the identification and biologic character of quiescent or slow-cycling tumor cells is needed.

In the present study, we used a membrane-bound dye, DiI, to identify slow-cycling cancer cells *in vitro *and *in vivo*. Our data directly confirm the existence of quiescent cells in growing colon tumor, and this cell population comprised only a small proportion of the tumor mass. Compared with other label-retention methods, DiI is simpler to use and yields easy identification of quiescent, label-retaining cells. However, it is important to note that the best time for analysis will differ depending on the type of tumor, because of the distinct proliferation cycle of different cells.

Many human cancers contain CSCs that are responsible for initiating and maintaining tumor growth and resistance to therapy [20-23]. The quiescent state seems to be necessary for preserving self-renewal of stem cells [24], and is a crucial factor in resistance to chemotherapy and targeted therapies [25-27]. In the present study, we used a tumor-forming assay to show the self-renewing potential of slow-cycling tumor cells *in vivo*. Simultaneous side-population analysis of the cell line indicated that CSCs were enriched in the slow-cycling population. It was particularly interesting that, although more transplanted tumors were seen in mice injected with slow-cycling tumor cells, the average tumor-forming time was longer than with the fast-cycling cells (Table 1, Table 2). This may because slow-cycling tumor cells take a long time to exit the quiescent state, and then expand and differentiate in response to stress. This finding indicates that, if the mechanism that causes recycling of quiescent cells could be elucidated and the crucial point of the pathway inhibited, this recycling could be inhibited, preventing tumor relapse and metastasis. Moreover, we found that, although the number of tumor cells and the volume of the tumor were reduced by drug treatment, the remnant was composed of drug-resistant, slow-cycling cells. These results provide evidence that slow-cycling tumor cells are resistant to traditional chemotherapy and are responsible for initiating tumor relapse and metastasis.

Conventional chemotherapy optimally targets highly proliferative tumor cells, and the existence of drug-resistant, slow-cycling tumor cells limits improvements in recurrence-free and overall survival rates. In this study, we found that drug-resistant tumor cells are mostly slow-cycling, and this population increased the proliferation of and IFN-γ production by spleen cells *in vitro*. Moreover, our *in vivo *experiments showed that, compared with normal tumor cells, vaccination with slow-cycling tumor cells generated a more effective immune response and prolonged the overall survival of tumor-bearing mice.

Although the slow-cycling population was more resistant to CTL cytotoxicity than the conventional tumor cells, this population could induce a more intense immune response, as shown by the enhanced cytotoxicity of spleen cells from mice immunized with slow-cycling tumor cells. More importantly, we found that these slow-cycling cells expressed a lower level of MHC class I molecules, but a higher level of class II, as well as a higher level of the co-stimulatory molecules CD80 and CD86, compared with conventional tumor cells. We speculate that the low expression of MHC class I molecules may have caused the resistance to killing by CTLs, whereas the upregulation of MHC class II and co-stimulatory molecules may be one reason for the increased induction of the immune response.

However, more questions remain about the mechanisms underlying the apparently superior outcomes from vaccination with slow-cycling tumor cells. For example, is there any difference between slow-cycling tumor cell antigens and conventional tumor lysates in inducing effector cell differentiation and memory T-cell generation? Further studies into different aspects of these tumor cells are needed. For instance, differences in gene expression between slow-cycling and conventional tumor cells have been analyzed by gene chip technology, and we have now found a series of overexpressed genes in slow dividing cells. One of these antigens, which has been reported to be a testicular cancer antigen, has particularly attracted our attention. However, further research into this gene and its related protein is needed.

The results of the present study all indicate that slow-cycling tumor cells are a better source of antigens for cancer immunization than conventional tumor cells. To date, the primary treatment for eliminating slow-cycling tumor cells is to induce them to enter the cell cycle and then kill them using traditional methods [2,28]. However, immunotherapy, as performed in our study, could selectively target the only slow-cycling tumor cells, resulting in elimination of the source of tumor recurrence and metastasis. Compared with conventional treatment, this technique could effectively reduce the risk of tumor recurrence and metastasis. Although several studies have shown that vaccination using stem-cell antigens induces a more effective immune response against prostate, brain, and ovarian cancers [29-31], there is controversy regarding the identification and isolation of CSCs in different tumors. Our results indicate that slow-cycling tumor cells could enrich CSCs, and the process we used to harvest slow-cycling tumor cells is easier to perform. Thus, the clinical application of this immunotherapy shows good prospects.

## Conclusions

In this study, we showed that slow-cycling tumor cells induced an antitumor immune response, especially of tumor-specific CTLs, with enhanced killing of drug-resistant tumor cells, and vaccination with slow-cycling tumor cells could prolong the overall survival of tumor-bearing mice. Our data also indicated that this treatment not only kills normal tumor cells, but also selectively targets the slow-cycling tumor cells, thus reducing the risk of cancer metastasis and relapse. Moreover, this vaccine has excellent histocompatibility, because slow-cycling tumor cells are isolated from the tissues of the recipient; thus, no severe side-effects should occur. To our knowledge, this is the first study of its kind. All our findings suggest that immunotherapy with inactivated slow-cycling tumor cells is a possible strategy to complement traditional cancer treatment.

## Abbreviations

5-FU: 5-Fluorouracil; Balb/C: a mouse strain; CFSE: 5- (and 6-) carboxyfluorescein diacetate succinimidyl ester; CSC: cancer stem cell; CTL: cytotoxic T lymphocyte; DiI,: 1'-dioctadecyl 3,3,3',3'-tetramethyl-indocarbocyanine perchlorate; DNase: deoxyribonuclease; ELISA: enzyme-linked immunosorbent assay; FACS: fluorescence-activated cell sorting; FBS: fetal bovine serum; FITC: fluorescein isothiocyanate; GM-CSF: granulocyte-macrophage colony-stimulating factor; HEPES: N-2-hydroxyethylpiperazine-N'-2-ethanesulfonic acid; IFN-g: interferon-g; MHC: major histocompatibility complex; MLTC: Mixed lymphocyte tumor cell culture; MMC: mitomycin-C; MTT: 3-(4,5-dimethylthiazol-2-yl)-2,5- diphenyltetrazolium bromide; NK cell: natural killer cell; PBS: phosphate-buffered saline; PE: phycoerythrin; PKH26: a red fluorescent dye; PI: propidium iodide; PY: Pyronin Y.

## Competing interests

The authors declare that they have no competing interests.

## Authors' contributions

SZ conceived of the study; SZ, QS and YZ participated in the design and coordination of the study; QS carried out the experiments, analyzed the data, and wrote the manuscript; FW performed the acquisition of data; CZ, DW, and WM carried out parts of the experiments and contributed to the guidance of experiments; and YH Z read the manuscript and revised it for important intellectual content. All authors have read and approved the final manuscript.

## Pre-publication history

The pre-publication history for this paper can be accessed here:

http://www.biomedcentral.com/1741-7015/10/172/prepub
